# *Polygonum multiflorum* Extracellular Vesicle-Like Nanovesicle for Skin Photoaging Therapy

**DOI:** 10.34133/bmr.0098

**Published:** 2024-12-19

**Authors:** Junjia He, Luoqin Fu, Yeyu Shen, Yan Teng, Youming Huang, Xiaoxia Ding, Danfeng Xu, Hong Cui, Mingang Zhu, Jiahao Xie, Yue Su, Ting Li, Weitao Huang, Xiaozhou Mou, Qiong Bian, Yibin Fan

**Affiliations:** ^1^ Center for Plastic & Reconstructive Surgery, Department of Dermatology, Zhejiang Provincial People’s Hospital, Affiliated People’s Hospital, Hangzhou Medical College, Hangzhou, Zhejiang 310014, China.; ^2^ Clinical Research Institute, Zhejiang Provincial People’s Hospital, Affiliated People’s Hospital, Hangzhou Medical College, Hangzhou, Zhejiang 310014, China.; ^3^ Department of Dermatology, the First People’s Hospital of Jiashan, Jiaxing, Zhejiang 314100, China.; ^4^ The Second Clinical Medical College, Zhejiang Chinese Medical University, Hangzhou, Zhejiang 310014, China.; ^5^ College of Bioengineering, Zhejiang University of Technology, Hangzhou, Zhejiang 310014, China.

## Abstract

Ultraviolet (UV) irradiation leads to the degradation of the extracellular matrix and collagen, thereby accelerating skin aging and imposing substantial psychological burden on patients. Current anti-aging strategies are limited and often associated with high costs or strong side effects. Plant-derived extracellular vesicle-like nanovesicles, with advantages such as natural availability and cost-effectiveness, show potential in anti-aging interventions. This study extracted extracellular vesicle-like nanovesicle from *Polygonum multiflorum* (PMELNVs) and systematically investigated their composition and metabolic pathways, further examining their efficacy and underlying mechanisms in combating photoaging. Results revealed the excellent antioxidative properties of PMELNVs, alleviating UV-induced oxidative stress, inhibiting matrix metalloproteinase production, reducing extracellular matrix degradation, promoting collagen synthesis, and ultimately exerting anti-photoaging effects. Additionally, safety assessments demonstrated favorable biocompatibility of PMELNVs. This study provides novel evidence supporting PMELNVs’ ability to resist photoaging by reducing oxidative stress and enhancing collagen expression, thereby offering potential as a new natural therapeutic agent against skin photoaging and promising a safer and more effective local anti-aging strategy.

## Introduction

The skin, as the body’s largest organ, serves as the primary natural barrier against external harmful substances, crucial for maintaining internal environmental stability. Skin aging not only compromises its protective function but also imposes significant psychological burden on individuals, making it a focal point of global research in recent years. Skin aging comprises both intrinsic and extrinsic factors, with external factors such as UV radiation, smoke, infrared radiation, malnutrition, and air pollution being the major contributing factors [1]. Exposure to UV radiation results in the generation of reactive oxygen species (ROS) by skin cells, which in turn leads to oxidative stress and inflammatory responses. ROS, as oxygen free radicals, can reversibly or irreversibly damage various compounds. UV radiation induces the synthesis of matrix metalloproteinases (MMPs) while inhibiting fibroblast function, leading to reduced collagen synthesis and secretion by fibroblasts. MMPs specifically degrade extracellular matrix components, disrupting the normal structure of collagen, ultimately resulting in skin photoaging [2–4]. Currently developed anti-aging strategies such as skincare regimens and the application of vitamin-based drugs suffer from unclear efficacy, high cost, or strong side effects [4–6]. Therefore, there is a urgent need for a natural, effective, and safe treatment with antioxidant properties for skin photoaging therapy, which holds significant scientific and clinical significance.

Extracellular vesicles (EVs) are membranous vesicular structures actively secreted by cells, ranging in size from 30 to 200 nm, and encapsulating proteins, lipids, nucleic acids, and other biologically significant molecules. They primarily originate from multivesicular bodies within cells and are subsequently released into the extracellular matrix following fusion of the outer membrane of the multivesicular body with the cell membrane [7–9]. Studies have shown that EVs, acting as active substances and carriers, play crucial roles in various skin-related fields including anti-aging [10]. EVs are primarily derived from animals and plants, with animal-derived EVs being utilized in skin anti-photoaging therapy and demonstrating favorable therapeutic effects [11,12]. However, limitations such as sourcing and low extraction yield hinder the application of animal-derived EVs. In recent years, plant-derived EVs extracted from roots, nuts, fresh and dried plants, etc., have been extensively explored for their advantages including safety, good biocompatibility [13], degradability without adversely affecting intestinal barrier function or causing toxicity to other organs, and scalability, making them promising for the treatment of various diseases [14–18]. Furthermore, some plant-derived EVs exhibit biological characteristics such as antioxidant and anti-inflammatory properties [18], making them a potential effective option for skin photoaging therapy.

*Polygonum multiflorum*, the dried tuberous root of *P. multiflorum*, is a traditional Chinese medicinal plant. As early as 973 AD, it was included in the “Kaibao Bencao”, a compendium of medicinal herbs compiled during the Song Dynasty under the decree of Emperor Taizu of Song. *P. multiflorum* products have traditionally been used for anti-aging, vitality enhancement, improving vascular health, darkening hair, strengthening bones, nourishing the liver and kidneys, and promoting longevity. Research has shown that anthraquinones, stilbene glycosides, proanthocyanidins, and 3,5,4-tetrahydroxystilbene-2-O-β-d-glucoside in *P. multiflorum* possess potent antioxidant and efficient free radical scavenger activities [19]. Previous studies have also indicated that processed *P. multiflorum* products can alleviate oxidative stress in epidermal cells, demonstrating potential in mitigating skin photoaging. However, issues such as the unclear effective ingredients and suboptimal effects of pure *P. multiflorum* extracts persist, highlighting the need for systematic research on its components and anti-photoaging treatment efficacy.

Therefore, in light of the above, we extracted *P. multiflorum* extracellular vesicle-like nanovesicle (PMELNVs) and conducted metabolomic sequencing to assess whether PMELNVs harbor relevant components and pathways for anti-skin photoaging. Both in vitro and in vivo experiments were utilized to assess the therapeutic efficacy and mechanisms of PMELNVs against skin photoaging. In vitro experiments demonstrated that PMELNVs enhanced the viability of human dermal fibroblasts (HDFs) under UV irradiation, reduced ROS production, and upregulated collagen expression while down-regulating MMP1 expression in HDFs. In vivo studies in a photoaged mouse model revealed that PMELNVs promoted wrinkle recovery and enhanced collagen deposition. The findings provide valuable insights into the development of effective anti-photoaging treatments and the potential of plant-derived EV-like nanovesicles in mitigating skin aging damage.

## Materials and Methods

### Isolation and culture of HDF cell line

The HDF cell line was obtained from Zhejiang Meisen Cell Technology Co. Ltd. and characterized using short tandem repeat (STR) analysis. The cells were incubated in a cell culture chamber at 37 °C with 5% CO_2_ using high-glucose Dulbecco’s modified Eagle’s medium (Vivacell, Germany), which was enhanced with a 10% addition of fetal bovine serum (Serana). The antibiotic penicillin concentration is 100 units/ml, while the streptomycin concentration is 100 μg/ml.

### Purification of PMELNVs

Fresh *P. multiflorum* was purchased and washed 3 times using water at ambient temperature. Subsequently, *P. multiflorum* is blended with a blender to make a juice. The obtained juice was filtered to remove large particles (EVital Bio, Hangzhou, Co. Ltd.). The filtrate was subjected to centrifugation at 3,000*g* for 30 min to remove larger particles and then underwent centrifugation at 10,000*g* for 60 min to eliminate microparticles. The supernatant obtained was subjected to centrifugation at 4 °C and a speed of 100,000*g* for a duration of 2 h. The pellet was reconstituted in sterile phosphate-buffered saline (PBS). The mixture was then further purified through the process of separation by centrifugal force 100,000*g* at 4 °C for 2 h to isolate PMELNVs. The protein concentration of purified PMELNVs was determined utilizing the BCA protein quantification assay kit (Thermo Fisher Scientific, USA). Purified PMELNVs were fixed with formaldehyde solution and treated with a blend of 1.8% methylcellulose and 0.4% uranyl acetate to achieve negative contrast staining. The sample was observed under an electron microscope, and microscopic photos were captured. The PMELNVs’ distribution of sizes was examined through dynamic light scattering (DLS) using a Zetasizer Nano ZS instrument (Malvern Instruments, UK).

### LC-MS analysis of PMELNV compounds

The liquid sample was obtained by using a solution of methanol and acetonitrile in a 1:1 ratio, with a volume of 390 μl. The combination was then subjected to sonication at a frequency of 40 kHz for a period of 35 min at a degree of heat of 5 °C. Following this, the samples were cooled to −40 °C for 40 min in order to induce protein precipitation. After subjecting the sample to centrifugation at 12,000*g* at 4 °C for 16 min, the liquid portion was cautiously moved to fresh microtubes and dried using a mild flow of nitrogen gas. For the ultrahigh-performance liquid chromatography–tandem mass spectrometry (UHPLC-MS/MS) analysis, the samples were reconstituted in 110-μl implementation of loading solution composed of equal parts of acetonitrile and water by volume (1:1, v/v), by briefly sonicating in a water bath at 5 °C. The extracted metabolites were centrifuged at 12,000*g* for 16 min at 4 °C on a bench-top centrifuge; subsequently, the supernatant was decanted and then placed into vials for analysis using LC-MS/MS.

The combination of the UHPLC system from Thermo Fisher Scientific with a Quadrupole-Orbitrap mass spectrometer, known as the UHPLC-Q Exactive system, was utilized for the LC-MS analysis in this study. The sample (2 μl) was subjected to separation using the HSS T3 column before being analyzed by mass spectrometry. The mobile phases were composed of water:acetonitrile (95:5, v/v) with 0.1% formic acid in solvent A and acetonitrile:isopropanol:an aqueous solution (47.5:47.5:5, v/v) containing 0.1% formic acid (solvent B). The change in slope of the solvent was adjusted under the following conditions: Starting from 0 to 0.11 min, there was a transition from 0% B to 5% B; then, from 0.11 to 2.1 min, it changed from 5% B to 25% B, followed by a shift from 2.1 to 9.1 min, going from 25% B to 100% B, maintaining at 100% B until the end of the period at 13.1 min; after that, there was a reversion back from 100% B to 0% B between 13.1 and 13.2 min; finally, for system equilibration, it remained at 0% B from 13.2 to 16.1 min. A 2-μl sample was injected at a flow rate of 0.39 ml/min, with the column temperature set at 4 °C. Throughout the timeframe for examination, the samples were kept in storage at 40 °C.

MS conditions: The Thermo UHPLC-Q Exactive Mass Spectrometer was utilized to gather mass spectrometric data, featuring an electrospray ionization (ESI) source that the operation can be conducted in either mode, whether positive or negative ion, and the optimal parameters were determined as follows: The heater was set to a temperature of 400 °C; capillary temperature was set at 320 °C; the gas flow rate through the sheath was maintained at 40 arbitrary units; auxiliary gas flow rate was kept at 10 arbitrary units; floating ion-spray voltage (FISV) was adjusted to −2,800 V when operating in the negative mode and 3,500 V when operating in the positive mode. Energy of impact was normalized at 20–40–60 V for MS/MS. The resolution for full MS was set at 70,000, while the resolution for MS/MS was 17,500. The data were obtained through the utilization of the mode of data-dependent acquisition (DDA). The identification was conducted throughout a mass range from 70 to 1,050 mass/charge ratio (*m/z*).

### Cell uptake of PMELNVs

PMELNVs were labeled with the Dil kit (Sigma-Aldrich) according to the manufacturer’s instructions. Briefly, the Dil dye was diluted with the provided diluent and added to PMELNVs for 30 min. The mixture was then transferred to an ultrafiltration tube and centrifuged at 110,000*g* (Beckman Coulter) for 1 h at 4 °C. After centrifugation, the labeled PMELNVs were filtered through a 0.22-μm filter. The Dil-labeled PMELNVs were co-incubated with HDF and examined using a Leica DMI8 laser confocal microscope.

### Cell viability assay

The biocompatibility and cytotoxicity of PMELNVs were assessed using the Cell Counting Kit-8 (CCK-8) colorimetric assay. HDF cells were seeded onto a concentration with a cell density of 8 × 10^3^ cells per well in a 96-well plate and incubated with PMELNVs diluted to final concentrations of 10, 20, 40, and 80 μg/ml, which were used for a duration of 24 h. The toxicity of PMELNVs on normal HDF cells was assessed employing the CCK-8 assay kit for cell viability assessment.

### Intracellular ROS generation assay

The production of ROS within HDF cells was identified using the Reactive Oxygen Species Assay Kit. HDF cells were plated in culture plates with a specified level of concentration of 100,000 cells per dish and left to incubate for 24 h. Aging was induced by UVB exposure, followed by treatment with 20 μg/ml of PMELNVs and further incubation at 37 °C for a duration of 24 h. The culture medium was exchanged using a new medium that includes 2′,7′-dichlorodihydrofluorescein diacetate (DCFH-DA) and subjected to incubation at 37 °C for a period of 20 min. The cells were ultimately marked with Hoechst 33342 for a duration of 20 min and subsequently examined using a Leica DMI8 laser confocal microscope.

### Levels of malondialdehyde, superoxide dismutase, lactate dehydrogenase, and senescence-associated β-galactosidase were assessed using assay kits to measure malondialdehyde and lactate dehydrogenase

The senescence-associated β-galactosidase (SA-β-gal) staining kit was employed to detect the positivity rate of senescent cells. The detection procedures were strictly conducted following the instructions provided with the assay kits.

### Mitochondrial membrane potential of HDF

The cells were seeded in laboratory containers with a compactness of 100,000 cells per dish and subjected to a 24-h incubation period. Aging was induced by UVB exposure, followed by treatment with 20 μg/ml of PMELNVs and further incubated at a temperature of 37 °C for a period of 24 h. The cells were subsequently subjected to culture medium containing JC-1 dye (10 μg/ml) for 15 min, followed by a wash with medium and subsequent incubation with fresh medium. The potential of the membrane in mitochondria was observed under a laser confocal microscope. JC-1 monomers were excited at wavelengths of 488 and 535 nm, while excitation of JC-1 aggregates occurred at 550 nm, with detection at 600 nm.

### Assessment of cellular viability

The HDF cell viability was assessed through the CCK-8 assay. HDF cells were seeded at a concentration of 8 × 10^3^ cells per well in a 96-well plate and cultured for 24 h. Aging was induced by UVB exposure, followed by treatment with varying levels of PMELNV concentrations (10, 20, 40, and 80 μg/ml) for 24 h. The viability of the cells was assessed with the CCK-8 assay kit.

### Analysis of Western blotting

The tissues and cells underwent processing in order to extract proteins suitable for 10% sodium dodecyl sulfate–polyacrylamide gel electrophoresis (SDS-PAGE) analysis and subsequently moved to preactivated polyvinylidene fluoride (PVDF) membranes. The membranes comprising proteins were obstructed using skim milk containing 5% fat and then were cultured in the presence of specific primary antibodies stored at 4 °C overnight: collagen type I (COL1) (1:1,000, Abclonal, China), collagen type III (COL3) (1:1,000, Abcam, UK), MMP-1 (1:1,000, Abcam, UK), and β-actin (1:50,000, Abclonal, China). After the tris-buffered saline with 0.1% Tween 20 detergent (TBST) wash, the membranes were exposed to secondary antibodies that were linked to horseradish peroxidase (1:5,000, Abclonal, China) at 37 °C for 1 h. Color development was achieved using an enhanced chemiluminescence solution, and images were acquired utilizing the gel imaging system from Bio-Rad. β-Actin was used as the reference protein in this study.

### Real-time quantitative analysis

The quantitative reverse transcription polymerase chain reaction (qRT-PCR) samples underwent lysis using Trizol reagent (Takara, Japan) to extract and quantify the total RNA concentration. Subsequently, 1,000 ng of RNA was converted into cDNA through reverse transcription utilizing the PrimeScript RT kit (Takara, Dalian, China). The resulting cDNA was exposed to instantaneous monitoring quantification using the ABI 7500 real-time fluorescence quantitative system (SYBR mix, Takara, Dalian, China). The reaction mixture was pre-denatured at 95 °C for 10 min, followed by 40 amplification cycles consisting of denaturation at 95 °C for 15 s, annealing at 60 °C for 1 min, and a melting curve stage. The 2^−ΔΔCT^ method was utilized to ascertain the relative levels of expression for the target genes, with each reaction conducted in triplicate. The expression values were normalized to β-actin for relative quantification. The primer sequences utilized in this investigation can be found in Table S1.

### Study of in vivo anti-photoaging effects of PMELNVs

The Animal Research Facility at Zhejiang Provincial People’s Hospital provided 5-week-old female nude mice for the study. Animal experimental procedures followed guidelines established by the Experimental Animal Committee at Zhejiang Provincial People’s Hospital. All experimental procedures involving animals were approved by the Ethics Committee of the Zhejiang Province People’s Hospital Animal House (approval number 20230725111419522049). The mice were divided into 4 groups in a random manner: control group, UVB irradiation group, PMELNV topical application group, and PMELNV subcutaneous injection group (20 μg/ml/2 d). Mice received daily UVB exposure 75 mJ/cm^2^ for 6 weeks, followed by topical application or subcutaneous injection of PMELNVs every other day for a 2-week treatment period. Digital photographs of the dorsal skin were taken at weeks 0, 3, 6, and 8. After completion of the study, the mice were humanely euthanized, and dorsal skin samples were collected for subsequent histological analysis. Each experimental group consisted of at least 5 mice.

### Tissue histopathological analysis

Following surgical excision, fresh nude mouse skin samples were preserved in a solution containing 4% PFA and subsequently embedded in paraffin wax. Paraffin-embedded sections (5 μm) were made in varying thickness and stained with hematoxylin and eosin (H&E). Histopathological changes in the skin sections were viewed using a light microscope. The paraffin-embedded sections underwent deparaffinization and rehydration for further analysis. Goat serum was used to avoid nonspecific attachment. The next step involves exposing the sample to primary antibodies, secondary antibodies, and the complex of avidin, biotin, and peroxidase. Samples were then incubated with diaminobenzidine (DAB) chromogen. The sections with stains were analyzed under a microscope, and quantification was performed using ImageJ software (version 1.54 d). Paraffin sections were further rehydrated, and 1.5% goat serum was used to block nonspecific binding. Following this, the sections were left to incubate for the duration of the night using primary antibodies at a temperature of 4 °C. After treatment with fluorophore-conjugated secondary antibodies, the pictures were examined with laser confocal microscopy. The ImageJ software was used to assess the fluorescence area and intensity.

### In vivo safety assessment of PMELNVs

To assess histological manifestations, a histological analysis was performed on tissue samples from the heart, liver, spleen, lung, and kidney. The sections were stained with H&E for examination. Hematological analysis and serum biochemical assays were conducted for blood routine examination.

### Statistical analysis

The statistical analysis was performed with the assistance of GraphPad Prism 9.0 software. The one-way analysis of variance (ANOVA) test was utilized for conducting comparisons across several different groups. Comparisons between 2 groups were conducted using the unpaired *t* test. The data are displayed in the format of mean ± SD. Statistical significance is denoted by *P < 0.05, **P < 0.01, ***P < 0.001, ****P < 0.0001.

## Results

### Characterization and identification of PMELNVs

**Fig. 1.**
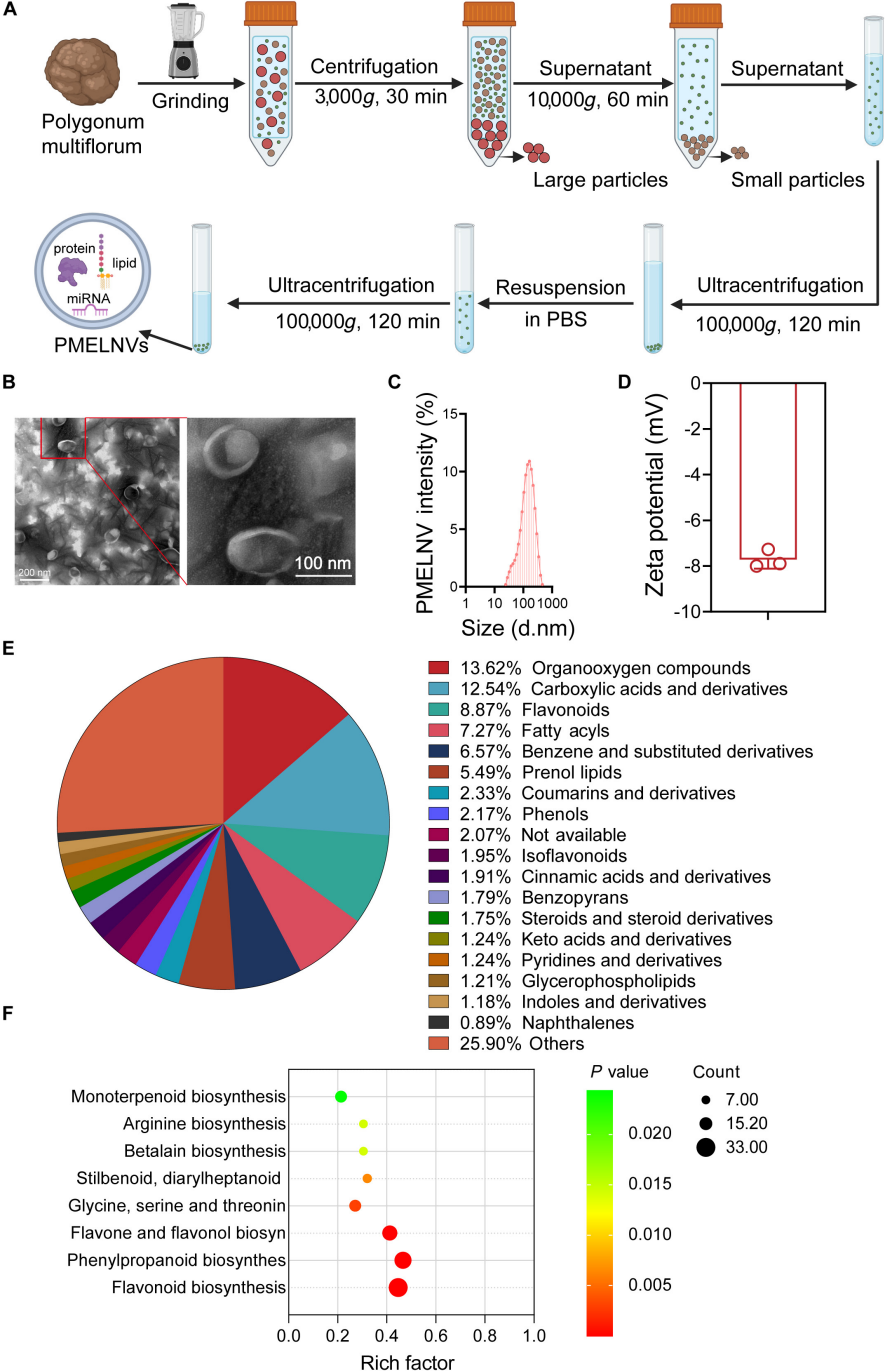
Detection and analysis of PMELNVs. (A) Schematic diagram illustrating the process of isolating and purifying PMELNVs. (B) Morphological characteristics and dimensions of PMELNVs following centrifugation using a sucrose gradient density characterized by TEM. Scale bar, 200 nm. (C) Dynamic light scattering analysis of the size distribution of PMELNVs. (D) Zeta potential of PMELNVs. (E) Identification of all compounds present in PMELNVs. (F) Enrichment analysis of KEGG pathways for all compounds present in PMELNVs.

To isolate PMELNVs from fresh *P. multiflorum*, the substance was processed into a homogeneous mixture with a juicer. The substance was processed into a homogeneous mixture with a juicer. The juice that was produced underwent filtration, and PMELNVs were isolated using a combination of various centrifugation techniques (Fig. 1A). Analysis of ultrastructure and morphology performed employing transmission electron microscopy (TEM) found that the vesicles in isolation displayed a characteristic rounded or cup-like morphology with discoidal concave structures, resembling EVs derived from mammalian cells (Fig. 1B). Furthermore, as depicted in Fig. 1C, the size of the nanovesicles predominantly ranged from 91.28 to 255.0 nm. This discovery was additionally reinforced through experiments using DLS. We did a Coomassie Brilliant Blue staining for PMELNVs and found that the main proteins were in the range of 15 to 20 kDa (Fig. S1). The average ζ potential of PMELNVs was −7.72 mV, indicating a negatively charged surface (Fig. 1D), which facilitates their uptake by skin cells. To gain deeper insights into the composition and potential biological functions of PMELNVs, comprehensive analyses were conducted using LC-MS technique. This examination uncovered the existence of a range of substances. Organic compounds constituted 13.62% of PMELNVs, followed by carboxylic acids and their derivatives (12.54%) and flavonoids (8.87%) (Fig. 1E). Characterizing the functionality of these substances revealed their significant enhancement of routes related to antioxidant stress, anti-aging metabolism, arginine iosynthesis, betalain biosynthesis, stilbenoid, diarylheptanoid and gingerol biosynthesis, glycine, serine, and threonine metabolism, monoterpenoid biosynthesis, flavone and flavonol biosynthesis, phenylpropanoid biosynthesis, and flavonoid biosynthesis (Fig. 1F). In summary, EV-like nanovesicle extracted from *P. multiflorum* exhibited characteristics similar to EVs, containing various antioxidant compounds and associated metabolic pathways, laying the foundation for further investigation.

### Cellular uptake of PMELNVs by HDFs, cytotoxicity of PMELNVs on HDFs, and antioxidant stress response

**Fig. 2.**
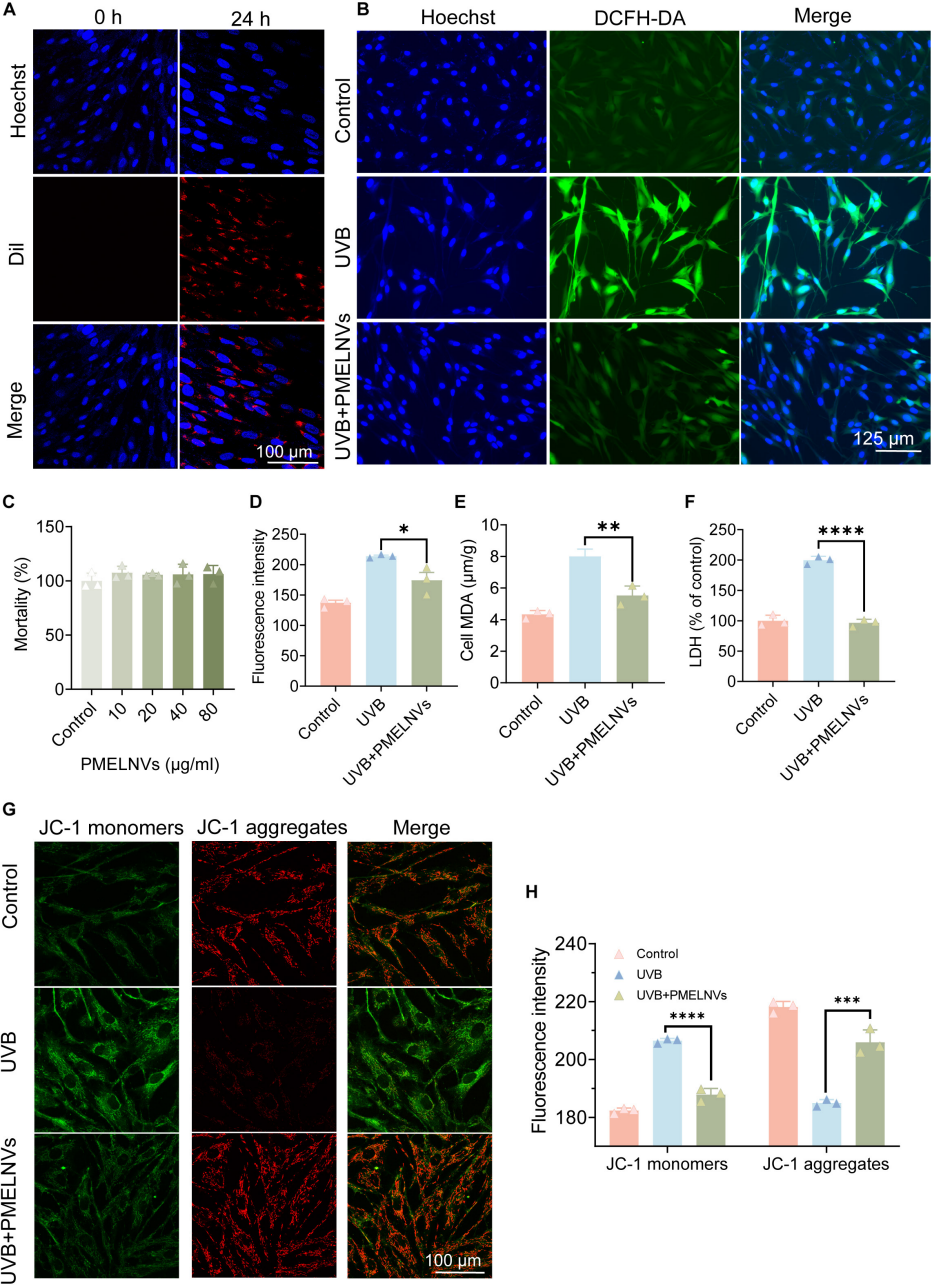
Cellular uptake of PMELNVs by HDFs and the antioxidant effect of PMELNVs in HDFs. (A) Uptake of PMELNVs by HDF cells. (B and D) PMELNVs reduce ROS production in UVB-damaged HDF cells and their quantification of fluorescence intensity. (C) Cytotoxic effect of PMELNVs on HDF cells. (E and F) PMELNVs reduce the production of MDA and LDH in UVB-damaged HDF cells. (G and H) JC-1 staining semiquantification of fluorescence images.

To assess the impact of PMELNVs on UV-exposed HDFs, we initially investigated the uptake of PMELNVs by HDFs. Dil-labeled PMELNVs were localized in the perinuclear region of HDFs (Fig. 2A). We monitored the cellular uptake of PMELNVs over time using confocal laser scanning microscope (CLSM) and flow cytometry, and did nano-flow detection of Dil-labeled PMELNVs (Fig. S2). The cytotoxicity of PMELNVs on HDF cells was evaluated by treating cells with different levels of PMELNV concentrations (10, 20, 40, and 80 μg/ml). In comparison to the control group, PMELNVs showed no significant toxicity to HDF cells (Fig. 2C). PMELNVs demonstrated the ability to reduce the expression of ROS in HDFs, as evidenced by a considerable reduction in the level of fluorescence emission of DCFH-DA compared to the UV-exposed group (Fig. 2B and D). We also detected the ROS using DCFH-DA assay by flow cytometry (Fig. S3). UVB radiation induced oxidative stress in HDFs, resulting in the generation of lipid peroxides. PMELNVs significantly reduced the concentrations of malondialdehyde (MDA), indicating a decrease in the process of lipid peroxidation (Fig. 2E). Cellular apoptosis or necrosis resulting in membrane structural damage leads to the liberation of enzymes from the culture medium containing cytoplasmic components, such as lactate dehydrogenase (LDH), released into it. Results showed that an elevation in LDH levels was also observed following UVB exposure, while PMELNVs significantly reduced LDH levels (Fig. 2F). Additionally, we analyzed the effect of PMELNVs on UV-damaged HDF cells by measuring the potential of the membrane in mitochondria. JC-1 fluorescent probe was employed for assessing the mitochondrial membrane potential in all experimental groups. When the mitochondrial membrane potential is elevated, JC-1 forms aggregates within the matrix of the mitochondria, forming polymers (J-aggregates), which emit red fluorescence. When the mitochondrial membrane potential is reduced, JC-1 is unable to form aggregates within the mitochondrial matrix, resulting in monomers, which emit green fluorescence. UV-induced damage to HDF cells significantly reduced mitochondrial membrane potential. However, the green fluorescence intensity in the PMELNV group was markedly reduced, indicating their capability to sustain the typical membrane potential under UV exposure (Fig. 2G and H).

### PMELNVs enhance HDF cell viability, reduce β-gal production, and upregulate COL1/3 to alleviate senescent cell activity

**Fig. 3.**
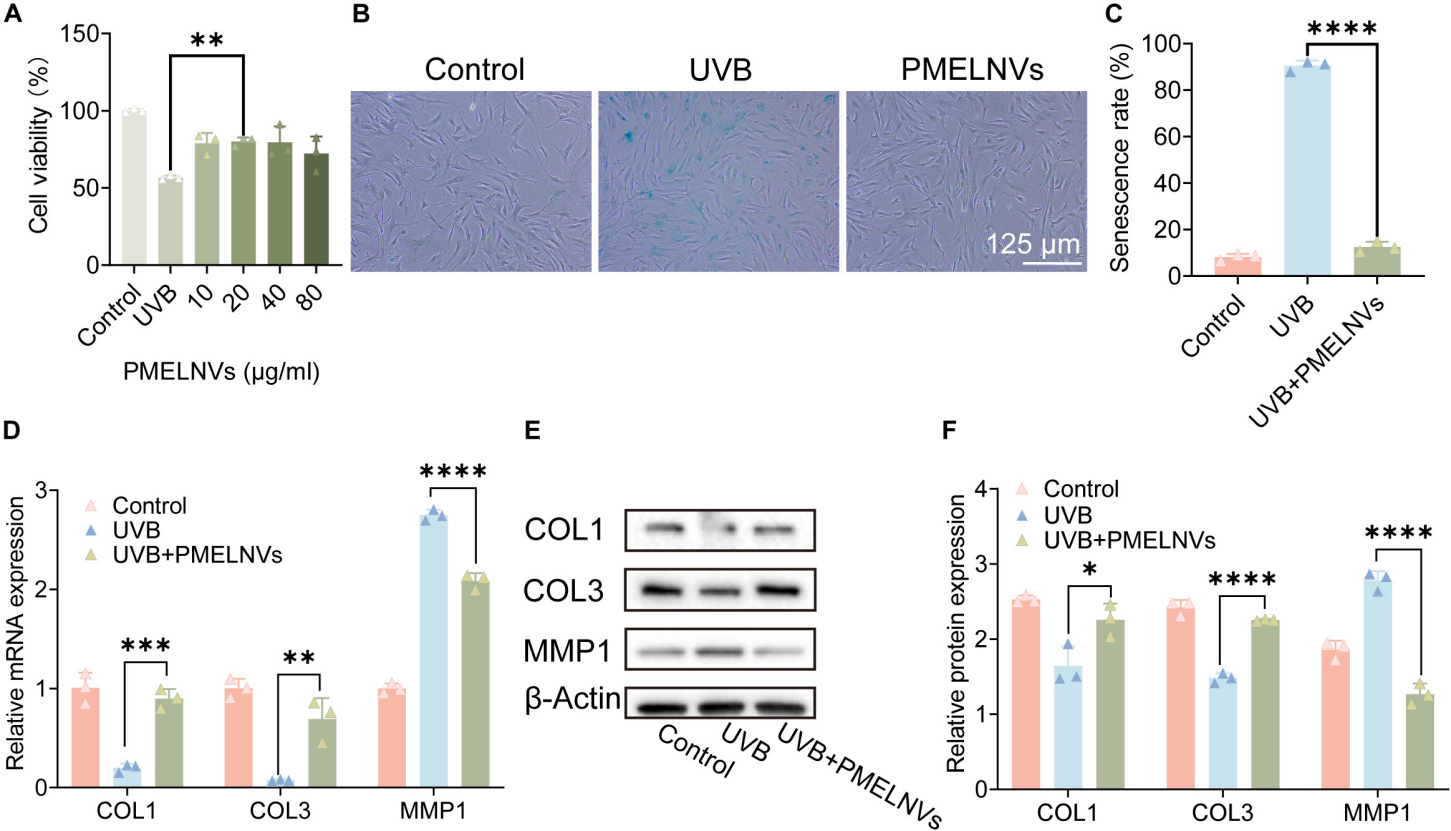
PMELNVs suppress β-gal and matrix metalloproteinase production, enhance HDF viability, and up-regulate collagen expression to counteract aging. (A) PMELNVs enhance the viability of UVB-damaged HDF cells. (B and C) β-Gal staining and its quantification data. (D) PCR examination of COL1, COL3, and MMP1 expression in HDF cells. (E and F) Analysis using the Western blot technique for COL1, COL3, and MMP1 expression in HDF cells and its quantification data.

PMELNVs are capable of enhancing the activity of UV-induced senescent HDF cells. Diverse levels of PMELNV concentrations (10, 20, 40, and 80 μg/ml) were applied to UV-induced senescent HDF cells. Compared to the UVB group, PMELNVs significantly enhanced the activity of UV-induced senescent HDF cells (Fig. 3A). Additionally, in the β-gal detection, the β-gal expression in HDFs treated with PMELNVs was assessed utilizing the SA-β-gal assay kit. It was observed that PMELNVs exhibited a down-regulatory effect on β-gal expression in HDFs, leading to a significant decrease in the percentage of aging cells (Fig. 3B and C). Furthermore, PMELNVs up-regulated the mRNA expression of COL1 and COL3 while down-regulating the expression levels of mRNA MMP-1 in HDFs (Fig. 3D). These changes were statistically significant in comparison with the UVB group (*P* < 0.05). Similar trends were noted at the protein level (Fig. 3E and F).

### PMELNVs exhibit anti-wrinkle and anti-aging effects in a photodamaged nude mouse model

One of the main challenges in studying skin photoaging using animal models lies in inherent differences in skin and subcutaneous tissue structure between humans and animals. Among various species used to simulate photoaging, nude mice are the most commonly employed. UV irradiation of the dorsal skin of nude mice is a widely accepted model for studying skin photoaging. This preference is partially attributed to the hairless nature of nude mice, which facilitates the observation of skin-specific changes induced by UV irradiation or subsequent treatments. Current research particularly focuses on examining structural and collagen arrangement alterations.

**Fig. 4.**
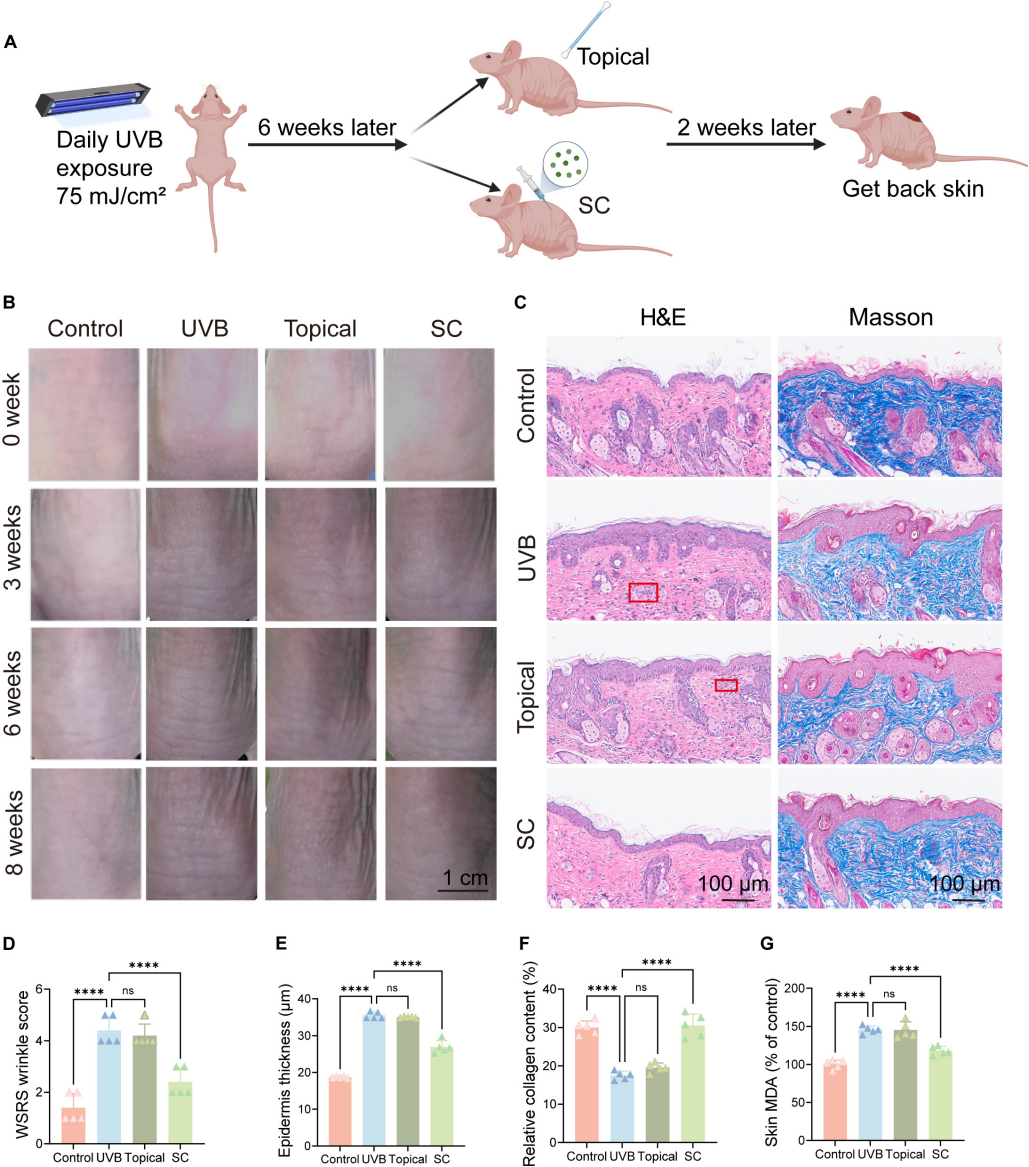
Effects of PMELNVs on the nude mouse photoaging model. (A) Schematic diagram of photoaging modeling in mice. (B) Changes in the dorsal skin of nude mice. (C) H&E and Masson staining. (D) Skin wrinkle scores. (E and F) Quantitative analysis of epidermal thickness using H&E staining and collagen deposition using Masson staining. (G) Skin MDA levels.

To examine the impacts of PMELNVs in an animal model, nude mouse dorsal skin was continuously exposed to UVB irradiation for 6 weeks to induce photoaging defects (Fig. 4A). Subsequently, PMELNVs were administered by subcutaneous injection or topical application. In vivo dose concentration was 20 μg/ml/2 d, and PMELNVs were labeled with Dir for distribution in vivo over time (Fig. S4). Digital photograph analysis revealed that subcutaneous injection of PMELNVs significantly promoted the recovery of skin wrinkles, while topical application did not produce noticeable effects, indicating inadequate skin penetration of PMELNVs (Fig. 4B). These results were obtained after 2 weeks of PMELNV treatment, with statistically significant reduction in skin wrinkles compared to pretreatment levels (*P* < 0.05) (Fig. 4D) according to the Wrinkle Severity Rating Scale [20] (Table S2). H&E staining and Masson’s trichrome staining were utilized to assess the thickness of the skin and the accumulation of collagen (Fig. 4C, E, and F). We also measured the relative content of MDA in the skin, and from Fig. 4G, it can be observed that subcutaneous injection of exosomes reduced MDA production. The findings indicated that the subcutaneous injection of PMELNVs increased dermal thickness and collagen fiber generation. This in vivo study further validates the anti-photoaging properties of PMELNVs and provides potential therapeutic strategies for clinical application.

### The role of PMELNVs in promoting collagen synthesis and attenuating oxidative stress in the photoaging nude mouse model

**Fig. 5.**
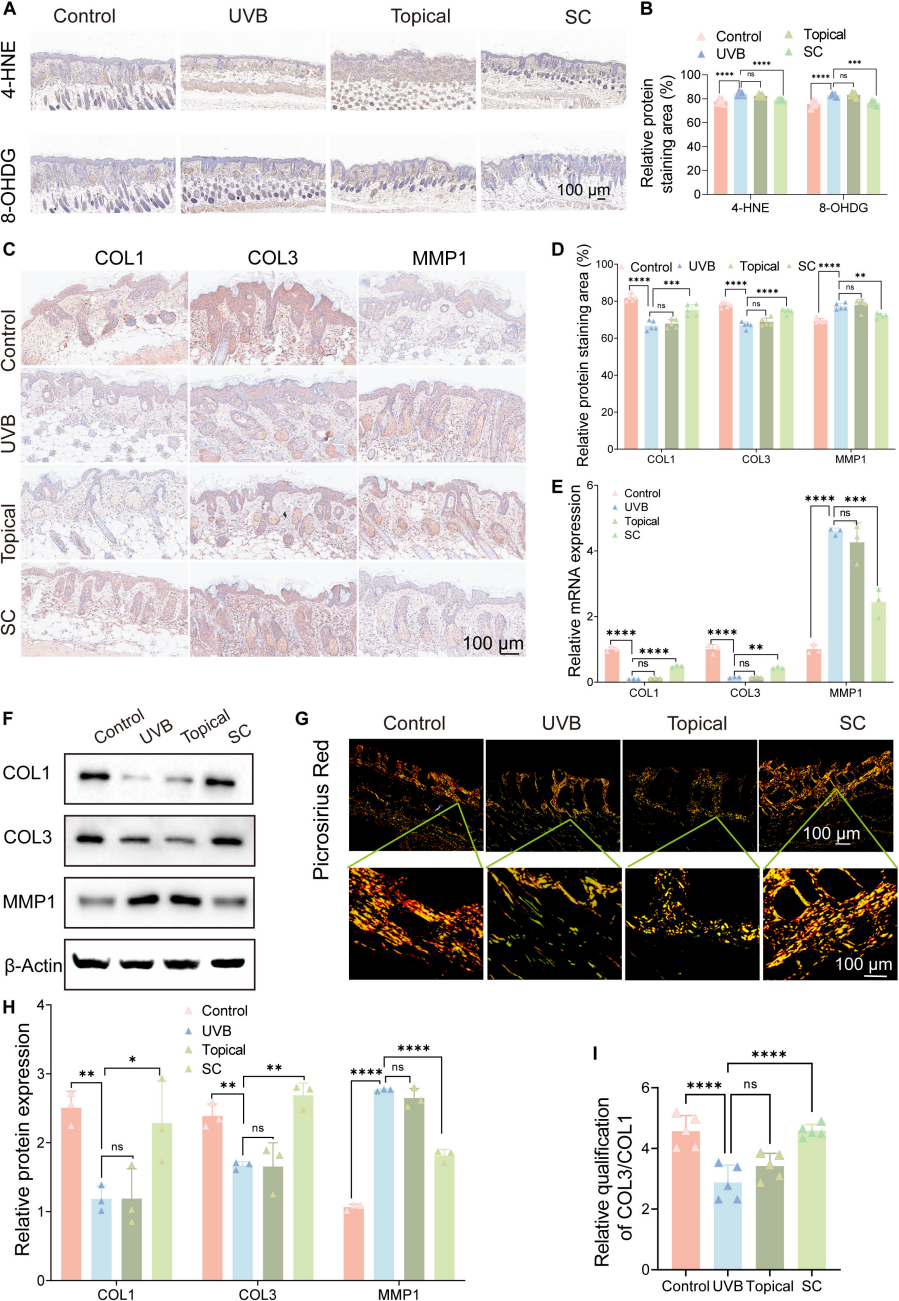
Evaluation of the anti-photoaging properties of PMELNVs in vivo. (A) Immunohistochemical detection of 4-HNE and 8-OHDG in the skin through staining. (B) Quantitative analysis of immunohistochemical detection of 4-HNE and 8-OHDG. (C) Immunohistochemical staining of COL1, COL3, and MMP1 in the skin. (E) mRNA expression (COL1, COL3, and MMP1). (F) Western blot analysis of COL1, COL3, and MMP1 in skin tissue. (D and H) Quantitative analysis of immunohistochemistry and Western blot. (G) Sirius Red staining with polarized light analysis. (I) Statistical data of Sirius Red staining for COL3/COL1.

To assess the influence of PMELNVs on the levels of oxidative stress in photoaging nude mice, skin tissue was collected for immunohistochemical examination of 4-hydroxynonenal (4-HNE) and 8-hydroxy-2′-deoxyguanosine (8-OHDG) expression levels. The results showed that subcutaneous injection of PMELNVs attenuated oxidative stress in the skin (Fig. 5A and B). Quantitative assessments of 4-HNE and 8-OHDG in skin tissue were determined using enzyme-linked immunosorbent assay (ELISA) and Western blot (Fig. S5). To study the effect of PMELNVs on collagen synthesis in the dermis of photoaging nude mice, skin tissue was obtained after 2 weeks of PMELNV treatment to assess the expression levels of collagen and MMP1. Subsequently, the collected skin tissue was analyzed to determine the expression of COL1, COL3, and MMP1. The results in Fig. 5C, D, F, and H showed that PMELNV treatment induced significant up-regulation of COL1 and COL3 expression and down-regulation of MMP1 expression. These results suggest that subcutaneous injection of PMELNVs promotes extracellular matrix remodeling in the dermis. The interpretation of the above results at the mRNA level is consistent (Fig. 5E). We stained collagen in the skin of nude mice with Sirius Red to assess the ratio of COL3/COL1, and the findings indicated that subcutaneous injection of PMELNVs increased the ratio of COL3 (Fig. 5G and I). These research findings further confirm the anti-photoaging properties of PMELNVs and their potential as a clinical treatment strategy.

### In vivo safety evaluation

We systematically evaluated the safety of PMELNVs in immunocompetent C57BL/6 mice (Fig. S6) and in nude mice, which is consistent with model mice.

**Fig. 6.**
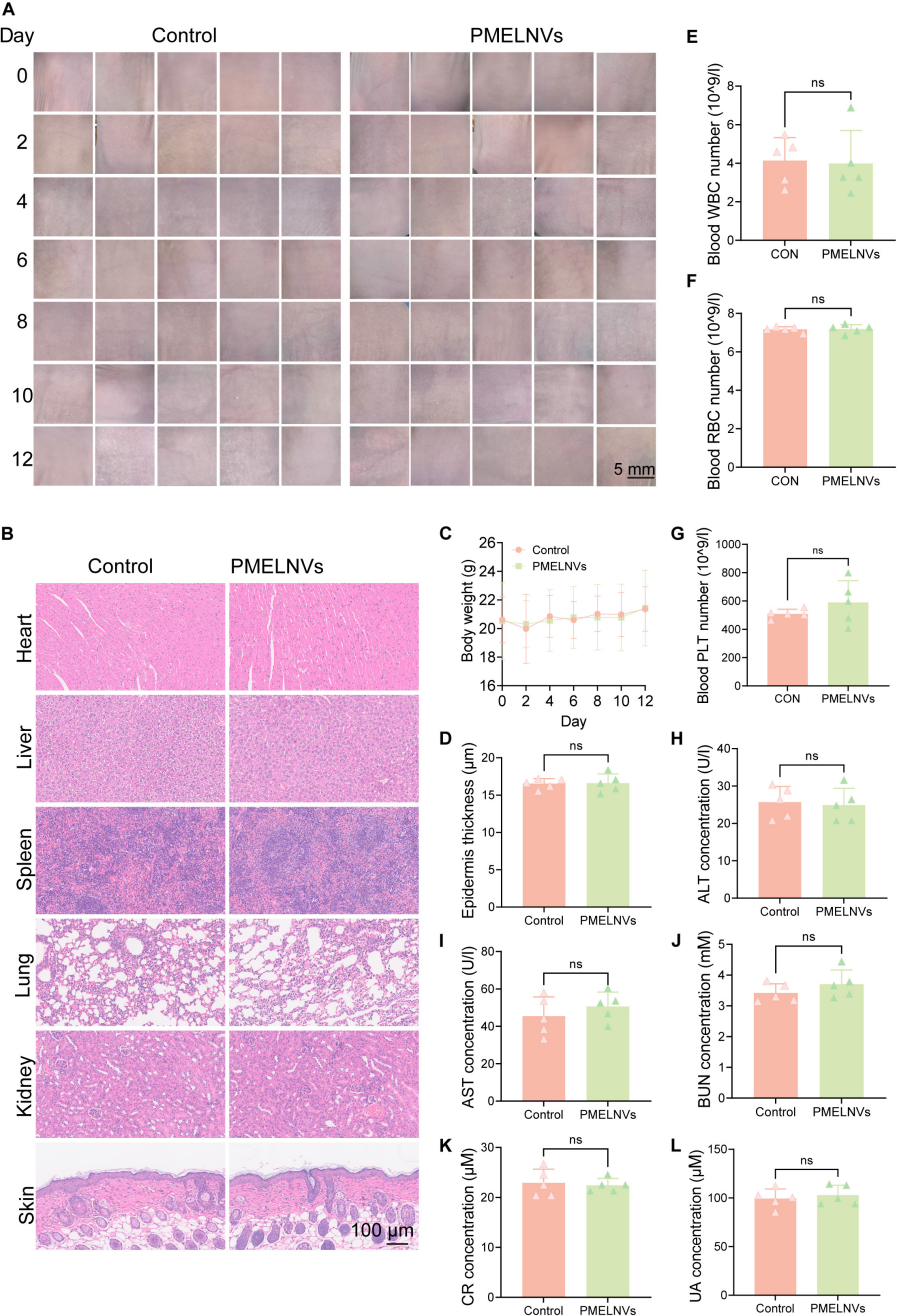
Safety evaluation of PMELNVs in vivo. (A) Skin condition before dosing every other day. (B and D) H&E-stained sections of major organs and skin tissue. (C) Changes in body weight. (E to G) Levels of RBC, WBC, and PLT. (H to L) Blood biochemical levels of ALT, AST, BUN, CR, and UA.

Every other day, digital images of the skin were captured. Digital images showed no significant changes in the skin of the PMELNV group compared to the normal control group (Fig. 6A). Pathological analysis of major organ tissue changes revealed that there were no notable pathological alterations detected in the primary bodily organs (heart, liver, spleen, lung, kidney) and skin of the PMELNV group compared to the normal control group (Fig. 6B and D). The distinction was lacking statistical significance in body weight between the PMELNV group and the normal control group (Fig. 6C). There were no significant differences in the white blood cell (WBC), red blood cell (RBC), and platelet (PLT) counts between the PMELNV group and the control group of normal nude mice regarding blood routine indicators (Fig. 6E to G). Regarding the impact on blood biochemical indicators, no substantial variances were observed in the levels of alanine aminotransferase (ALT), aspartate aminotransferase (AST), blood urea nitrogen (BUN), serum creatinine (CR), and serum uric acid (UA) among the PMELNV group and the normal control group (Fig. 6H to L).

## Discussion

The aging of skin can be attributed to various factors, among which ultraviolet (UV) radiation is the primary cause, accounting for about 80% of skin aging. Existing research indicates that UVB can promote the formation of ROS and other photoproducts associated with skin cancer and aging, such as MMPs. Upon exposure to UV radiation, various signaling pathways responsible for promoting photoaging and skin cancer are activated in skin cells, regulating cell growth, proliferation, and survival, leading to changes in skin. In dermal fibroblasts and other dermal cells, UV radiation inhibits the synthesis of type I collagen, while the overexpression of MMPs leads to a decrease in the content of collagen and elastin, degrading the structural proteins that maintain skin elasticity.

Plant-derived extracellular vesicle-like nanovesicles (PELNVs), as alternatives to mammalian cell-derived exosomes, have gained increasing popularity among researchers, surpassing past constraints on efficiency. Because of their innate source and composition, along with the lack of harmful microorganisms that affect humans, PELNVs have advantages undetectable by the immune system: high safety in the human body, long circulation period, and enhanced bioavailability. Moreover, PELNVs exhibit significant stability of physicochemical properties in different surroundings, enabling them to resist degradation through the action of enzymes in the human digestive system. The role of EVs in the skin has also been reported, such as EVs derived from *Lactobacillus plantarum*, inducing anti-inflammatory M2 macrophage polarization in vitro, regulating skin immunity, and exerting anti-inflammatory effects [21].

This study extracted EVs from the dried roots of Polygonaceae plant *P. multiflorum*, which possess a unique double-layered shell structure, potentially imparting better payload capacity and stability. The protective potential of PMELNVs against skin photoaging was investigated. Previous research has reported that *P. multiflorum* extract exhibits anti-aging, hepatoprotective, hematopoietic, hypolipidemic, and anti-atherosclerotic effects. Through metabolomic analysis and Kyoto Encyclopedia of Genes and Genomes (KEGG) pathway enrichment analysis of PMELNVs, it was found that the metabolome contains a significant proportion of antioxidant stress-related metabolites, with flavonoids, which have both free radical scavenging and antioxidant properties, being prominent. Additionally, KEGG pathway enrichment analysis revealed significant enrichment of antioxidant stress-related metabolic pathways in the PMELNV metabolome.

Subsequently, the anti-aging effects of PMELNVs were studied using UVB-induced in vitro and in vivo aging models. The findings indicated that PMELNVs exhibited significant antioxidant stress reduction in UVB-induced aging HDFs, significantly reducing ROS production and mitochondrial membrane potential, decreasing oxidative stress in UVB-induced HDF, reducing LDH production, decreasing lipid oxidation, and reducing the expression of the aging phenotype β-gal. PMELNVs also suppressed the level of expression of MMP1, enhanced the level of COL1 and COL3, reduced UVB-induced oxidative stress, and increased collagen production. In vivo, by reducing oxidative stress-induced damage to the skin and increasing the ratio of COL3 to COL1, PMELNVs achieved therapeutic effects in a nude mouse photoaging model. Safety experiments demonstrated no local or systemic toxicity.

Therefore, PMELNVs, as a novel natural therapeutic agent against photoaging skin, show promising potential for clinical treatment.

## Data Availability

The datasets supporting the conclusions of this article are included within the article and its additional file.
