# Supplementary material for: Polygonum multiflorum Extracellular Vesicle-Like Nanovesicle for Skin Photoaging Therapy
Source: Biomater Res. 2024 Dec 19;28:0098. doi: 10.34133/bmr.0098 (PMC11658808; doi:10.34133/bmr.0098)
Supplement: Supplementary 1 — Tables S1 and S2 Figs. S1 to S6 [file bmr.0098.f1.docx]

|  | Gene symbols | Primer Sequence |
| --- | --- | --- |
| mouse | COL1A1-FORWARD | AGGCGAACAAGGTGACAGAGG |
|  | COL1A1-REVERSE | GGAGAACCAGGAGAACCAGGAG |
|  | COL3A1-FORWARD | CGTGGCTCTAATGGCATCAAAGG |
|  | COL3A1-REVERSE | ATGTGGTCCAACTGGTCCTCTG |
|  | MMP1-FORWARD | CAGTTGACAGGCTCCGAGAAATG |
|  | MMP1-REVERSE | CACATCAGGCACTCCACATCTTG |
|  |  |  |
| Human | COL1A1-FORWARD | ICCAAAGGGIGACAAGGGIGAAC |
|  | COL1A1-REVERSE | PGGAGGACCAATAGGACCAGTAGG |
|  | COL3A1-FORWARD | EGGTGAACCTGGTGCTCCTG |
|  | COL3A1-REVERSE | IGCTCCTOGCTTCCTTCCTCTC |
|  | MMP1-FORWARD | KGCTGCTGCTGCTGTTCTGG |
|  | MMP1-REVERSE | CAACTTGCCTOCCATCATICTTCAG |

Table S1 Primer Sequence

Table S2 The Wrinkle Severity Rating Scale

| Score | Category | Description |
| --- | --- | --- |
| 1 | Absent | Fold not visible; continuous line of skin. |
| 2 | Mild | Superficial fold, but visible and with a mild depression. |
| 3 | Moderate | Moderately deep fold, less than 1 mm deep. |
| 4 | Severe | Very long and deep fold, less than 2 mm deep. |
| 5 | Extreme | Long and extremely deep fold, 2 to 4 mm deep, producing an older facial aspect. |


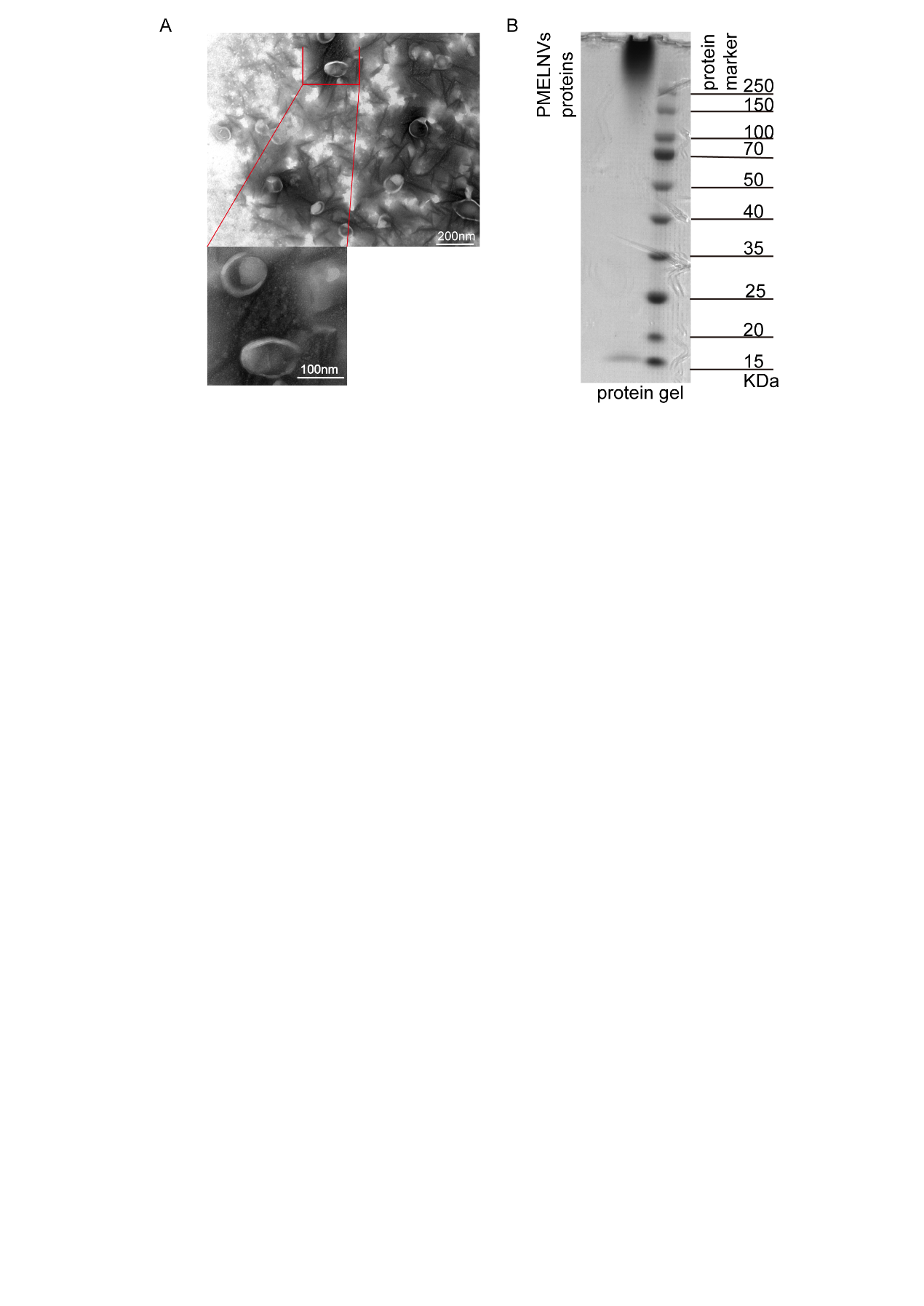
Fig. S1. Coomassie Brilliant Blue staining for PMELNVs.


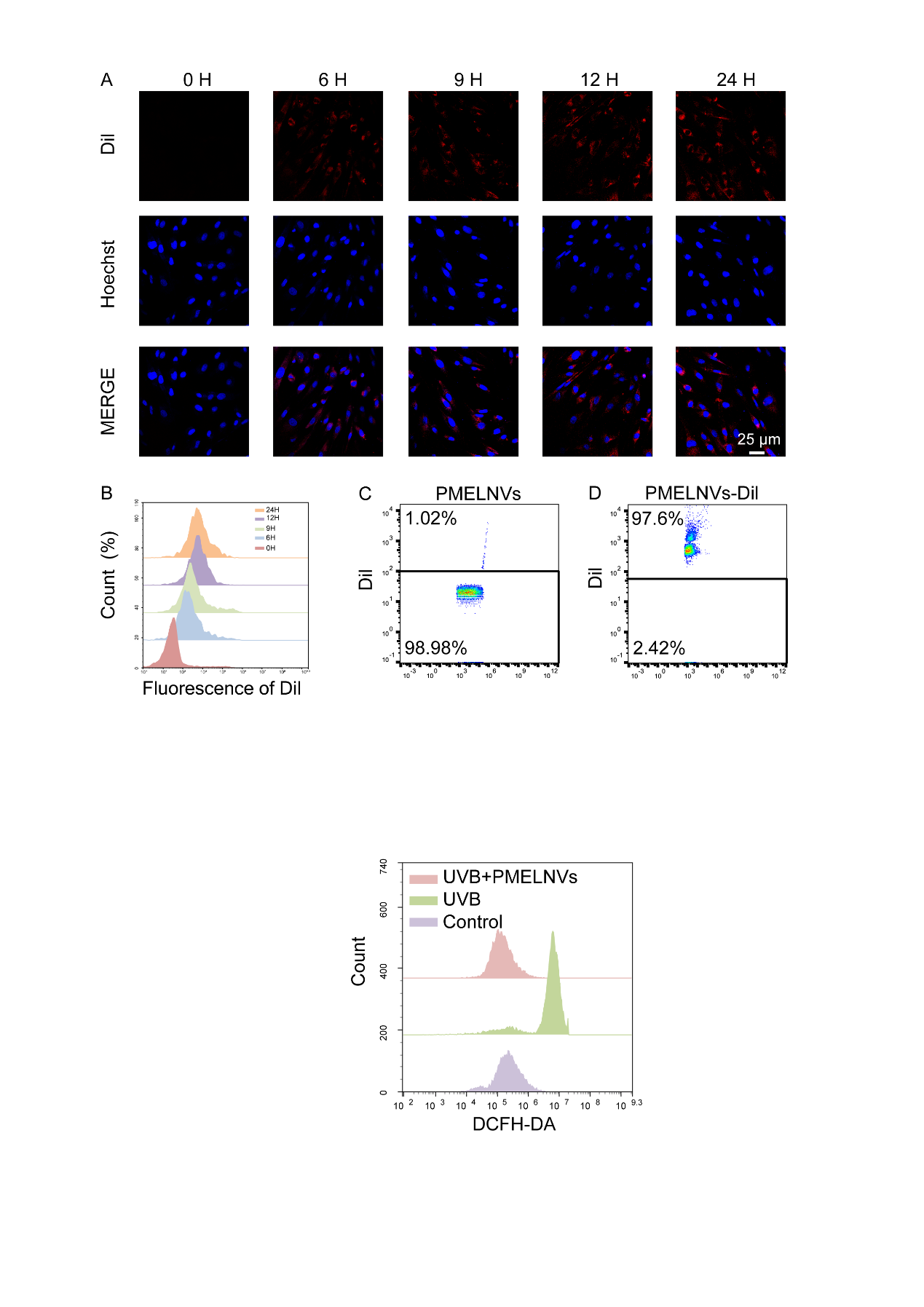


Fig. S2. (A) Cellular uptake of PMELNVs over time. (B) Flow cytometry of cellular uptake over time. (C, D) Nano-flow detection of Dil-labeled PMELNVs.


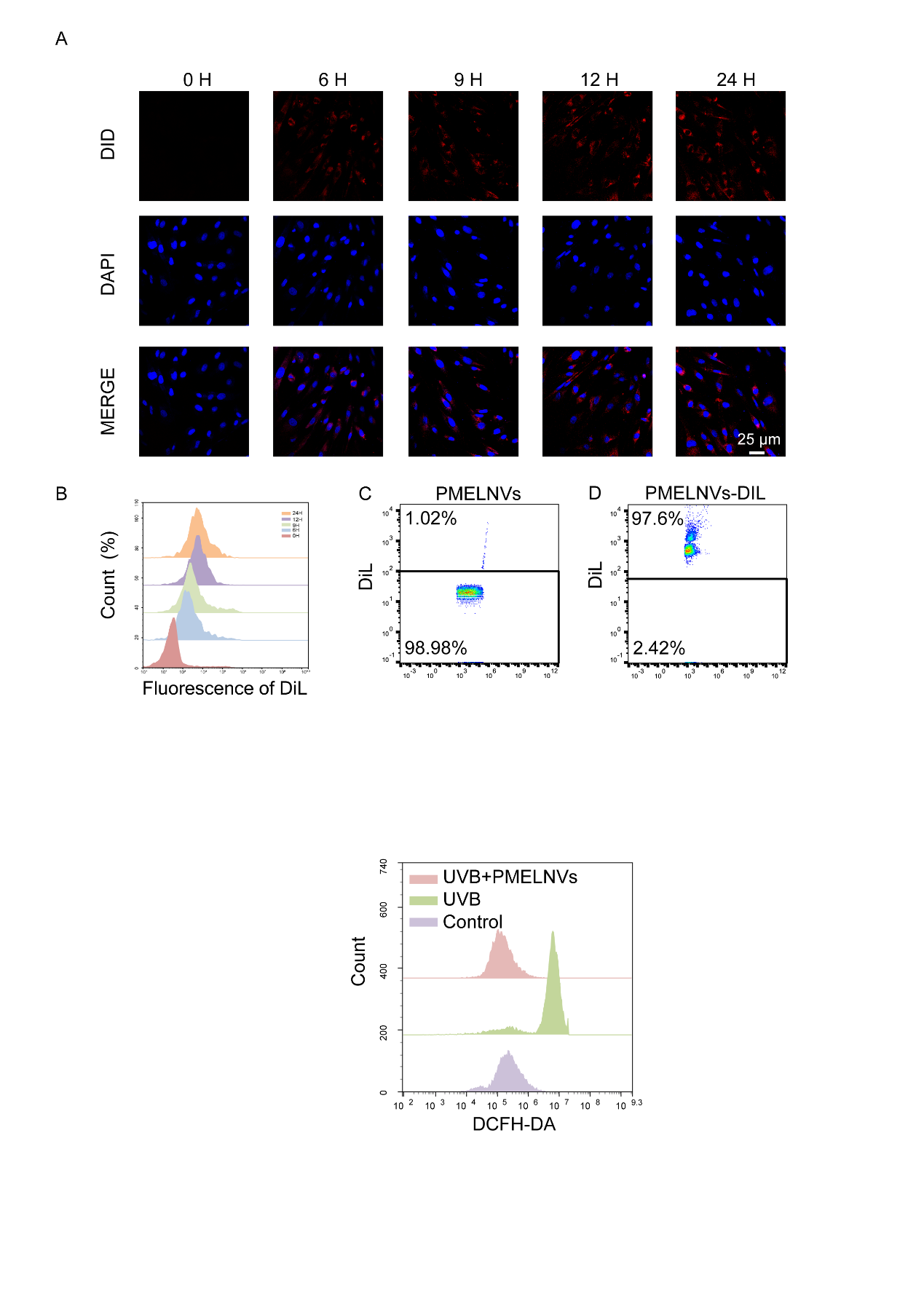


Fig. S3. ROS detection by flow cytometry.


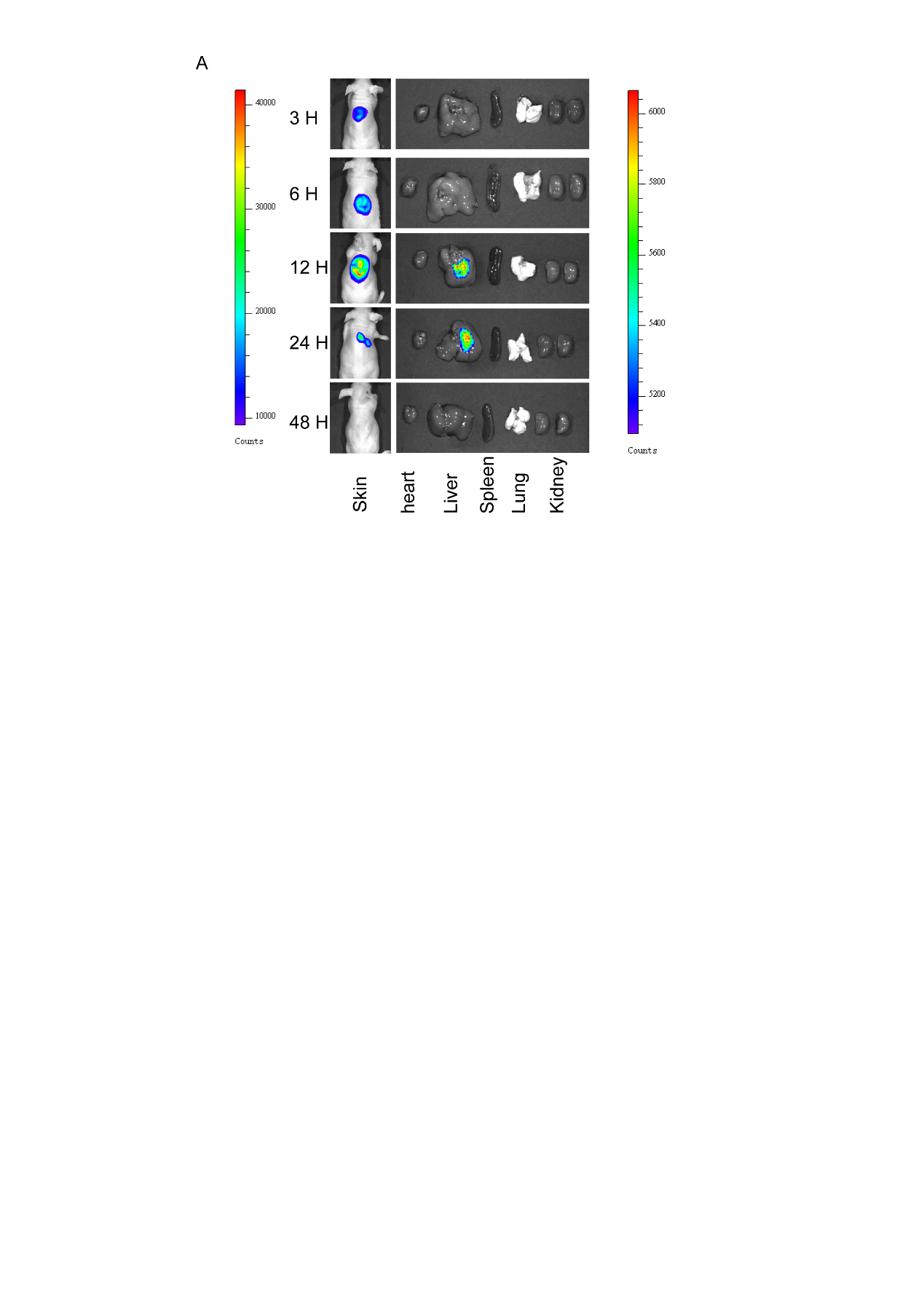
Fig. S4. *In vivo* distribution of PMELNVs after S.C. administration.


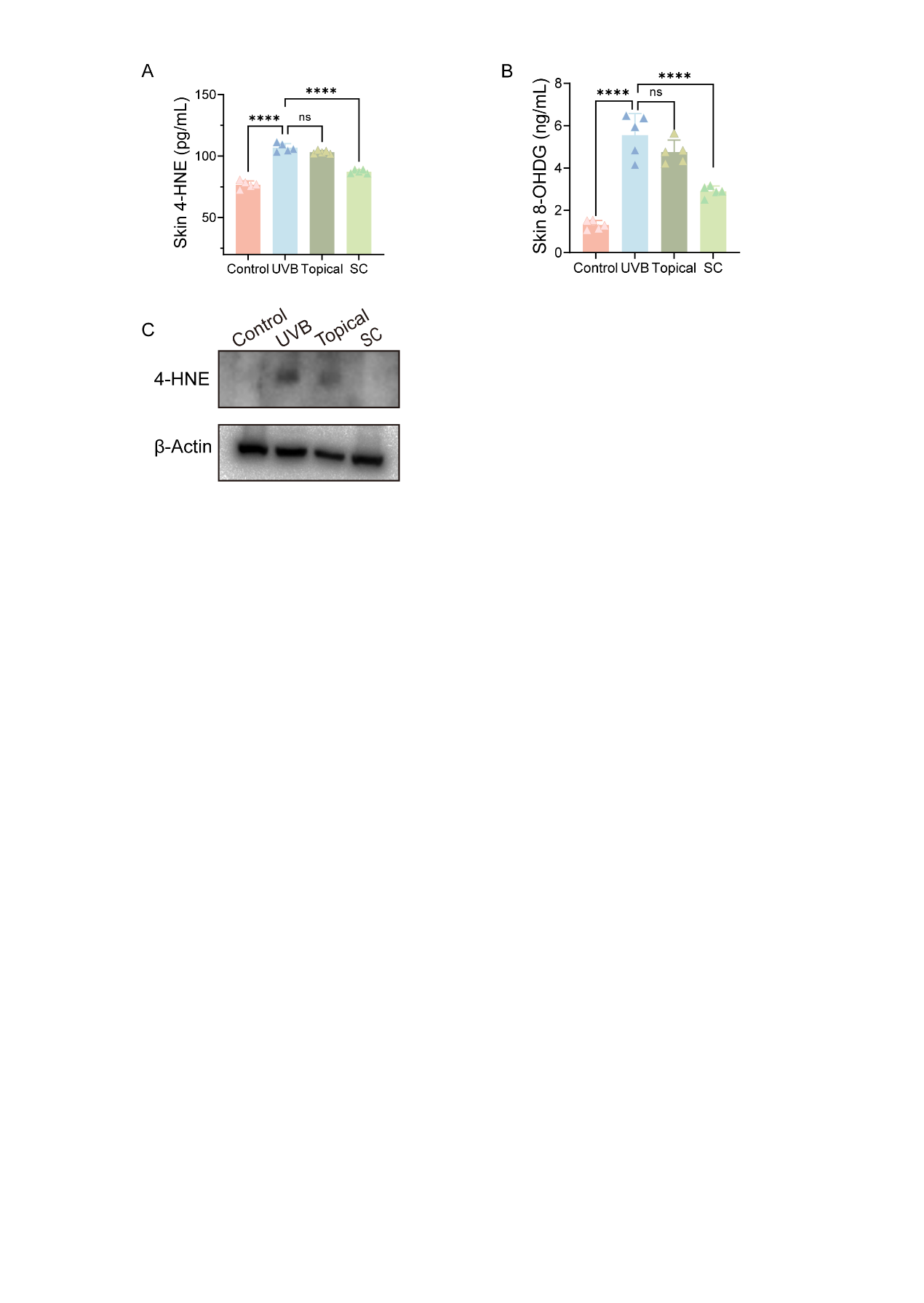


Fig. S5. (A, B) Elisa results for 4-HNE and 8-OHDG amounts in skin tissue. (C) 4-HNE in skin tissues by Western blot.


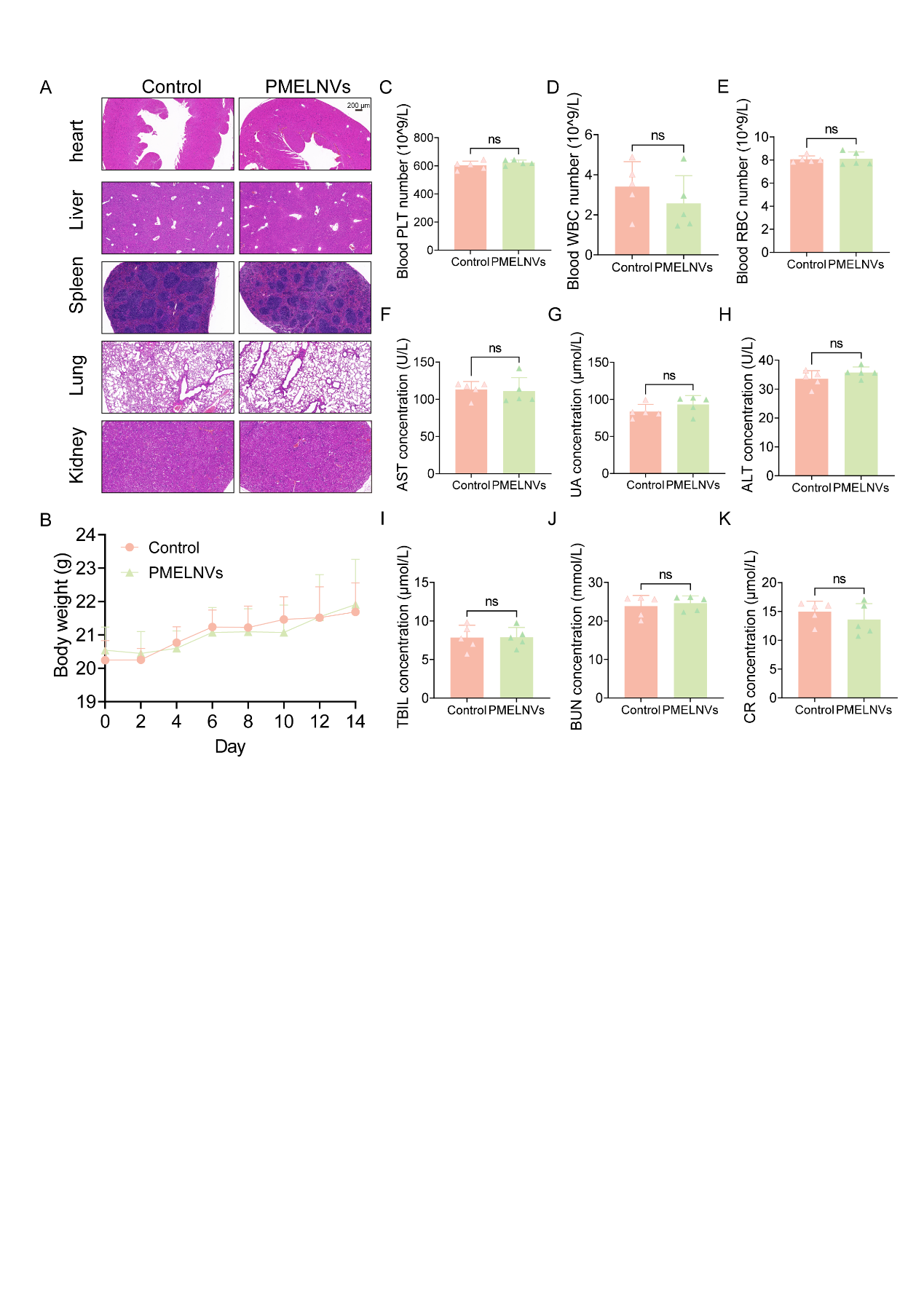


Fig. S6. (A) HE-stained sections of major organs. (B) Changes in body weight. (C, D, E) Levels of PLT, WBC, RBC. (F, G, H, I, J, K) Serum biochemical levels.
